# Higher Grip Strength Is Associated With Reduced Risk of Incident Symptomatic Hand Osteoarthritis: Data From Two Cohort Studies

**DOI:** 10.1002/jcsm.70265

**Published:** 2026-03-31

**Authors:** Yilun Wang, Wei Li, Hongyi He, David John Hunter, Weiya Zhang, Michael Doherty, Yuqing Zhang, Junqing Xie, Yanqiu Zhu, Jiatian Li, Tuo Yang, Jie Wei, Guanghua Lei, Chao Zeng

**Affiliations:** ^1^ Department of Orthopaedics Xiangya Hospital, Central South University Changsha China; ^2^ Rheumatology Department, Royal North Shore Hospital and Institute of Bone and Joint Research, Kolling Institute University of Sydney Sydney Australia; ^3^ Academic Rheumatology, School of Medicine University of Nottingham Nottingham UK; ^4^ Pain Centre Versus Arthritis University of Nottingham Nottingham UK; ^5^ Division of Rheumatology, Allergy, and Immunology, Department of Medicine Massachusetts General Hospital, Harvard Medical School Boston USA; ^6^ The Mongan Institute Massachusetts General Hospital, Harvard Medical School Boston USA; ^7^ Nuffield Department of Orthopaedics, Rheumatology and Musculoskeletal Sciences University of Oxford Oxford UK; ^8^ Key Laboratory of Aging‐related Bone and Joint Diseases Prevention and Treatment, Ministry of Education Xiangya Hospital, Central South University Changsha China; ^9^ Hunan Key Laboratory of Joint Degeneration and Injury Xiangya Hospital, Central South University Changsha China; ^10^ Health Management Center Xiangya Hospital, Central South University Changsha China; ^11^ Department of Epidemiology and Health Statistics, Xiangya School of Public Health Central South University Changsha China; ^12^ FuRong Laboratory Changsha China; ^13^ National Clinical Research Center for Geriatric Disorders Xiangya Hospital, Central South University Changsha China

**Keywords:** cohort, grip strength, hand osteoarthritis, Mendelian randomization

## Abstract

**Background:**

Grip strength is increasingly recognized as a modifiable and easily measurable protective factor against chronic diseases. Lower grip strength may compromise joint stability, predisposing periarticular tissues to local inflammation, which has been implicated in hand osteoarthritis (HOA), a condition affecting approximately 189 million people globally and imposing substantial health and socio‐economic burden. Whether lower grip strength is a risk factor for clinically relevant symptomatic HOA remains unclear. We aimed to examine the association between grip strength and incident symptomatic HOA to inform targeted preventive strategies.

**Methods:**

We conducted prospective cohort studies and Mendelian randomization analyses using data from the Xiangya Osteoarthritis (XO) Study and UK Biobank. Individuals without baseline symptomatic or hospital‐diagnosed HOA were included. Genetic instruments for grip strength were derived from genome‐wide association studies. Symptomatic HOA in the XO Study was defined as the presence of radiographic HOA with symptoms, whereas hospital‐diagnosed HOA in the UK Biobank was ascertained through hospital inpatient records.

**Results:**

Among 2869 XO Study participants (5461 hands; 55.9% women; mean age of 63.2 years), 166 (3.0%) hands developed incident symptomatic HOA during a mean follow‐up of 3.7 years. Compared with the lowest grip strength quartile, odds ratios (ORs) and their corresponding 95% confidence intervals (95% CIs) of symptomatic HOA in the second, third and highest quartiles were 0.51 (95% CI, 0.31–0.84), 0.62 (95% CI, 0.39–0.99) and 0.46 (95% CI, 0.28–0.75), respectively (*p* for trend = 0.003). Similar associations were observed among 481 582 UK Biobank individuals (54.2% women; mean age of 56.4 years). Mendelian randomization analyses showed ORs of genetically determined grip strength of 0.56 (95% CI, 0.40–0.78, *p* < 0.001) for incident symptomatic HOA in the XO Study and 0.37 (95% CI, 0.25–0.56, *p* < 0.001) for incident hospital‐diagnosed HOA in the UK Biobank.

**Conclusions:**

Higher grip strength was associated with a lower risk of incident symptomatic HOA. These findings offer empirical evidence that interventions aimed at enhancing grip strength may help prevent symptomatic HOA and reduce its individual and societal burden.

## 1 Introduction

Grip strength, an easy and non‐invasive method to assess muscle strength, has been identified as a robust marker of sarcopenia and is associated with adverse health outcomes such as all‐cause mortality [1, 2]. Its significance is increasingly acknowledged as a potentially modifiable protective factor against various chronic diseases [3, 4]. Grip strength is typically measured using handheld dynamometers, which are available in hydraulic, pneumatic, and electronic models. Among these, the Jamar dynamometer is considered the gold standard due to its high reliability and precision, particularly in geriatric populations [5, 6, 7].

Hand osteoarthritis (HOA), affecting approximately 189 million people worldwide [8], imposes a substantial burden on individuals and society due to pain, dysfunction of the hand and reduced quality of life [9]. Despite its impact, our understanding of risk factors for HOA remains incomplete, leading to limited interventions to prevent or mitigate its effect [10]. Identifying modifiable and easily implementable risk factors could guide targeted strategies to alleviate the burden of HOA. Lower grip strength could impact the stability of joints, consequently leading to localized inflammation in the periarticular tissues, which have been implicated in HOA [11]. However, whether lower grip strength is a risk factor for HOA and enhancing grip strength has the potential to prevent the occurrence of HOA or modify its course remains unclear.

Previous studies have investigated the association between grip strength and HOA, and the findings remain inconclusive. The early evidence, mainly based on cross‐sectional studies, consistently showed that individuals with radiographic HOA exhibit reduced grip strength [12, 13, 14]. The results from the Framingham Osteoarthritis Study, a prospective cohort study, found that higher grip strength was associated with an increased incident radiographic HOA [15]. Nevertheless, a substantial proportion of participants in that study did not have repeated hand radiographs at the follow‐up examination, making the results susceptible to potential selection bias. The findings from the subsequent two cohort studies were inconsistent: one showed that higher grip strength increased the risk of radiographic HOA progression in men [16], whereas another did not observe such an association [17]. Because both studies were conducted among individuals with prevalent HOA, the results may be affected by potential collider bias [18]. To date, there is a paucity of evidence of the causal association between grip strength and incident symptomatic HOA. Considering that symptomatic HOA, rather than radiographic HOA alone, correlates with functional limitations, disability, and health care utilization [19], elucidating the association between grip strength and symptomatic HOA has significant clinical and public health implications.

**FIGURE 1:**
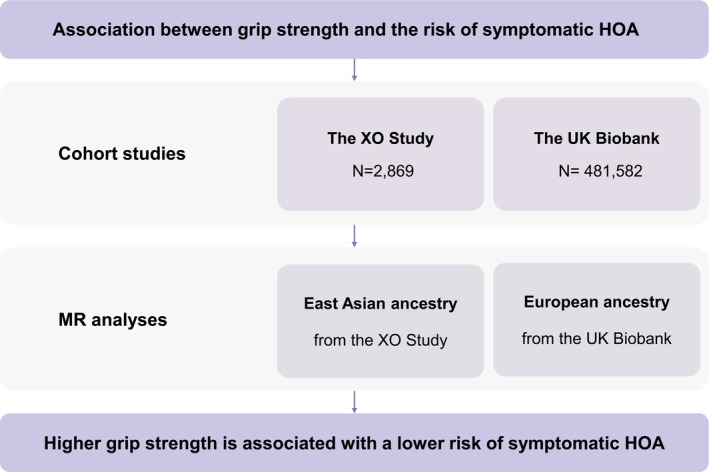
Overview of study design. HOA, hand osteoarthritis; MR, Mendelian randomization; XO Study, Xiangya Osteoarthritis Study.

To address this knowledge gap, we examined the association between grip strength and incident symptomatic HOA in the Xiangya Osteoarthritis (XO) Study, a general population‐based cohort conducted in China [20, 21], and replicated the findings using data from the UK Biobank [22]. To minimize the residual confounders and strengthen the causal inference, we employed Mendelian randomization (MR) analyses to examine the relation between genetically determined grip strength and incident symptomatic HOA in the XO Study and hospital‐diagnosed incident HOA in the UK Biobank (Figure 1).

## 2 Materials and Methods

### 2.1 Study Design

This study utilized data from two large, population‐based cohorts in China and the United Kingdom, combining prospective cohort analyses with MR approaches. All analyses and reporting were conducted in accordance with the Strengthening the Reporting of Observational Studies in Epidemiology (STROBE) statement [23] and the Strengthening the Reporting of Mendelian Randomization Studies (STROBE‐MR) guidelines [24].

The XO Study is a population‐based prospective cohort study investigating the natural history and risk factors of OA. Individuals aged ≥ 50 were randomly selected from rural mountainous villages in Longshan County in Hunan Province, China (NCT04033757). Details of the cohort have been described previously [20, 21]. Briefly, the study consists of three sub‐cohorts (i.e., sub‐cohorts I, II and III), which were initiated in 2015 (*n* = 1469), 2018 (*n* = 1271) and 2019 (*n* = 1340), respectively. The Ethics Committee of Xiangya Hospital, Central South University (201510506) approved the study. Written informed consent was obtained from all participants.

The UK Biobank is a cohort study that enrolled over 500 000 participants aged 37–73 at baseline between 2006 and 2010 [22]. All participants completed a questionnaire, underwent physical measurements and provided blood samples at baseline. The present research was approved by the UK Biobank Research and Access Committee (11/NW/0382).

### 2.2 The XO Study

#### 2.2.1 Participants

**FIGURE 2:**
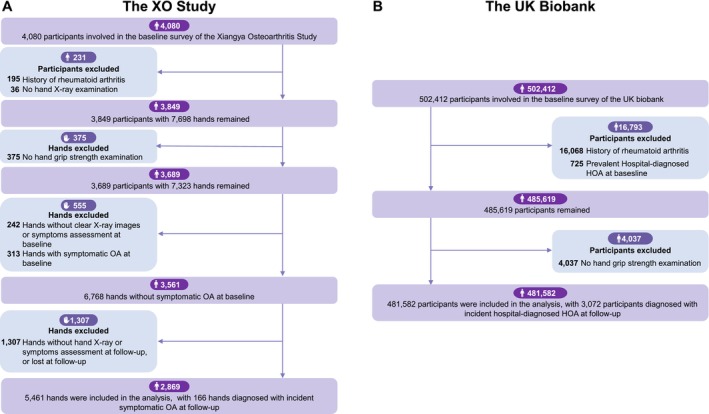
Selection process of the included participants for incident symptomatic/hospital‐diagnosed HOA. HOA, hand osteoarthritis; XO Study, Xiangya Osteoarthritis Study.

As shown in Figure 2, participants in the XO Study with complete handgrip strength measurements, hand x‐ray images and hand symptom assessments were included in the analysis. We excluded hands with symptomatic HOA at baseline, those lacking x‐ray or symptom assessments at baseline or during follow‐up and those lost to follow‐up. Participants who self‐reported rheumatoid arthritis at baseline were also excluded.

#### 2.2.2 Assessment of Symptomatic HOA

All participants had bilateral hand radiographs using the postero‐anterior view at baseline and each year during the 3‐year follow‐up. Using a modified Kellgren–Lawrence (KL) grade (ranging from 0 to 4), two readers evaluated and graded the radiographs of the following 15 joints bilaterally: carpometacarpal 1, metacarpophalangeal 1–5, proximal interphalangeal 1–5 and distal interphalangeal 2–5 (eMethods). Hand symptoms were ascertained by a participant's response to the question, ‘on most days, do you have pain, aching, or stiffness in your left/right hand?’ If they answered ‘yes’, a homunculus was shown to identify symptomatic joint(s) [25]. Incident symptomatic HOA was defined as the diagnosis of symptomatic HOA based on both signs of radiographic evidence of OA (KL grade ≥ 2 in any of the joints of each hand) and the presence of symptoms in the same hand at any follow‐up visit in a participant who did not meet the corresponding criteria at baseline [26].

#### 2.2.3 Grip Strength Measurement

Grip strength was measured using a calibrated Jamar 5030J1 hydraulic hand dynamometer with participants sitting. Three measurements, taken at 10‐s intervals for each hand, were averaged to determine the final grip strength for each hand. Inter‐/intra‐class correlation coefficients were good for both left/right hand grip strength (eMethods). Subsequent hand‐based cohort analyses considered left/right hand grip strength separately, whereas an average of final left/right hand grip strength was used for each participant in person‐based analyses and MR analyses.

#### 2.2.4 Covariates

Demographic information (age, sex, education and hand injury history) was collected during face‐to‐face interviews by trained professionals. Body mass index (BMI) was calculated as weight (kg) divided by the square of height (m^2^). All covariates were assessed on the same day as the HOA evaluation.

#### 2.2.5 Statistical Analyses

We divided grip strength into sex‐ and age‐specific quartiles (age was grouped in 2‐year intervals). We calculated the total and sex‐specific incident symptomatic HOA over the follow‐up period for each quartile of grip strength. The primary analysis was conducted at the hand level using generalized estimating equations, adjusting for age, sex (men, women), education (yes, no), BMI (< 18.5 kg/m^2^, 18.5–24 kg/m^2^, 24–28 kg/m^2^, ≥ 28 kg/m^2^) and history of hand injury (yes, no). Dose–response associations were assessed using restricted cubic spline models, with prespecified knots at the 5th, 10th, 50th and 90th percentiles of grip strength. Sex‐stratified analyses were also performed. Sensitivity analyses involved a person‐based analysis using logistic regression to examine grip strength and incident symptomatic HOA. To minimize potential reverse causality bias (pain or radiographic HOA reduces grip strength), we excluded participants with only radiographic HOA or hand pain at baseline or adjusted radiographic HOA or hand pain at baseline in the multivariable regression model, respectively.

### 2.3 The UK Biobank

#### 2.3.1 Participants

Similarly in the UK Biobank, participants with complete data on handgrip strength and hospital‐diagnosed HOA were included in the analysis. Hands with hospital‐diagnosed HOA at baseline and participants with rheumatoid arthritis, identified using the International Classification of Diseases, Ninth Revision (ICD‐9) codes or Tenth Revision (ICD‐10) [27], were excluded.

#### 2.3.2 Diagnosis of Hospital‐Diagnosed HOA

HOA in the UK Biobank was identified through hospital inpatient records using the ICD‐9 codes or ICD‐10, along with the date of first inpatient diagnosis [28]. Incident hospital‐diagnosed HOA was defined as the diagnosis of HOA after the study baseline [29]. Because HOA in the UK Biobank was classified by hospital physicians, these cases are expected to represent clinically symptomatic cases [28].

#### 2.3.3 Grip Strength Measurement

Grip strength at the study baseline was measured using a Jamar J00105 hydraulic hand dynamometer with participants sitting upright, following standard procedures for reliable and reproducible measurements [30]. One measurement per hand was recorded. Given the person‐based diagnosis of hospital‐diagnosed HOA, a single average of left and right hands was used for each participant in subsequent analyses [31].

#### 2.3.4 Covariates

Age was determined from the date of birth to the baseline assessment. Sex and education were self‐reported at baseline. BMI was the calculated measure of weight/height^2^ available in the anthropometrics data at recruitment. Hand injury history was diagnosed through hospital inpatient records using ICD‐9 or ICD‐10 codes, along with the date of first inpatient diagnosis.

#### 2.3.5 Statistical Analyses

Participants were classified into four groups according to sex‐ and age‐specific quartiles (age was grouped in 2‐year intervals) of the baseline grip strength, with the lowest quartile as the reference group. We performed Cox proportional hazard models to examine the relation of grip strength to the incident hospital‐diagnosed HOA, accounting for the competing risk of death using the Fine–Gray method, adjusting for age, sex, BMI, education and history of hand injury. Sex‐stratified analyses were also performed. We used restricted cubic splines to smooth the dose–response relation of grip strength to the rate of incident hospital‐diagnosed HOA. To minimize potential reverse causality bias, we implemented 1‐year and 2‐year exposure lag times by excluding HOA cases that occurred within 1 or 2 years after the index date to evaluate the robustness of our study findings.

Missing data were handled using multivariate imputation by chained equations [32]. A *p* value < 0.05 (two‐sided) was considered statistically significant. Statistical analyses were conducted using SAS 9.4 (SAS Institute, Cary, NC, USA) and R software 4.2.2 (R Foundation for Statistical Computing, Vienna, Austria).

### 2.4 MR Studies

MR analyses were conducted to investigate potential causal associations between grip strength (exposure) and symptomatic HOA (outcome) in the XO Study or hospital‐diagnosed HOA (outcome) in the UK Biobank. The validity of MR analyses relies on three key assumptions [33]: (1) The IVs are associated with the exposure (grip strength); (2) the IVs are independent of the confounders; and (3) the IVs affect the outcome (HOA) only through the exposure (grip strength).

#### 2.4.1 Whole‐Genome Sequencing in the XO Study

A total of 2980 blood samples that passed quality control in the XO Study were sequenced using BGI's DNBSEQ platform. Raw data generated from the DNBSEQ platform were then filtered with default parameters in fastp software for quality control and aligned to the reference panel GRCh38, with an average effective depth of 38.4 × (minimum 21.8×) and an average coverage of 99.2% (minimum 98.7%). GATK was used for single‐nucleotide polymorphism (SNP) and insertion–deletion (indel) calling, which finally generated 54 916 001 high‐quality markers. Further sequencing details were presented in the eMethods.

#### 2.4.2 Genotyping in the UK Biobank

DNA was purified from biological samples obtained during the initial assessment. The initial 49 950 participants were genotyped using the UK BiLEVE Axiom array, and the remaining 438 427 participants were genotyped using the closely related UK Biobank Axiom array. These arrays share 95% common content, covering approximately 800 000 SNPs and indel markers. Quality control and imputation were performed centrally by the Wellcome Trust Centre for Human Genetics.

#### 2.4.3 Statistical Analyses for MR Studies

We first conducted genome‐wide association studies (GWASs) in the XO Study to calculate summary‐level genetic data (β coefficients and standard errors) for grip strength and incident symptomatic HOA. A one‐sample MR study was then conducted using SNPs associated with grip strength (*p* < 1 × 10^−5^). Pruning involved pairwise linkage disequilibrium with an *R* ^2^ threshold of > 0.001, with the linkage disequilibrium reference panel specific to individuals with East Asian ancestry for statistical independence. Causal estimates (odds ratios [ORs]) were generated using the inverse‐variance weighted (IVW) method as the primary analysis, supplemented by several sensitivity methods that make different assumptions, including MR Egger, weighted median and MR pleiotropy residual sum and the outlier (MR‐PRESSO) test. *F*‐statistics were utilized to assess the strength of the instrument variables (IVs), whereas Cochran's *Q* test was used to assess the heterogeneity. Details of the GWAS and MR analyses are in the eMethods. Except for the reference panel (specific to individuals with European ancestry) used to prune SNPs in linkage disequilibrium, other procedures and statistical methods implemented in the UK Biobank were the same as the XO Study. A *p* value < 0.05 (two‐sided) was considered statistically significant. Statistical analyses were conducted using SAIGE Version 1.1.9; GCTA Version 1.94.1; and R software Version 4.2.2 (R Foundation for Statistical Computing, Vienna, Austria) with the TwoSampleMR package (Version 0.5.7).

## 3 Results

### 3.1 Association Between Grip Strength and Incident Symptomatic HOA in the XO Study

**FIGURE 3:**
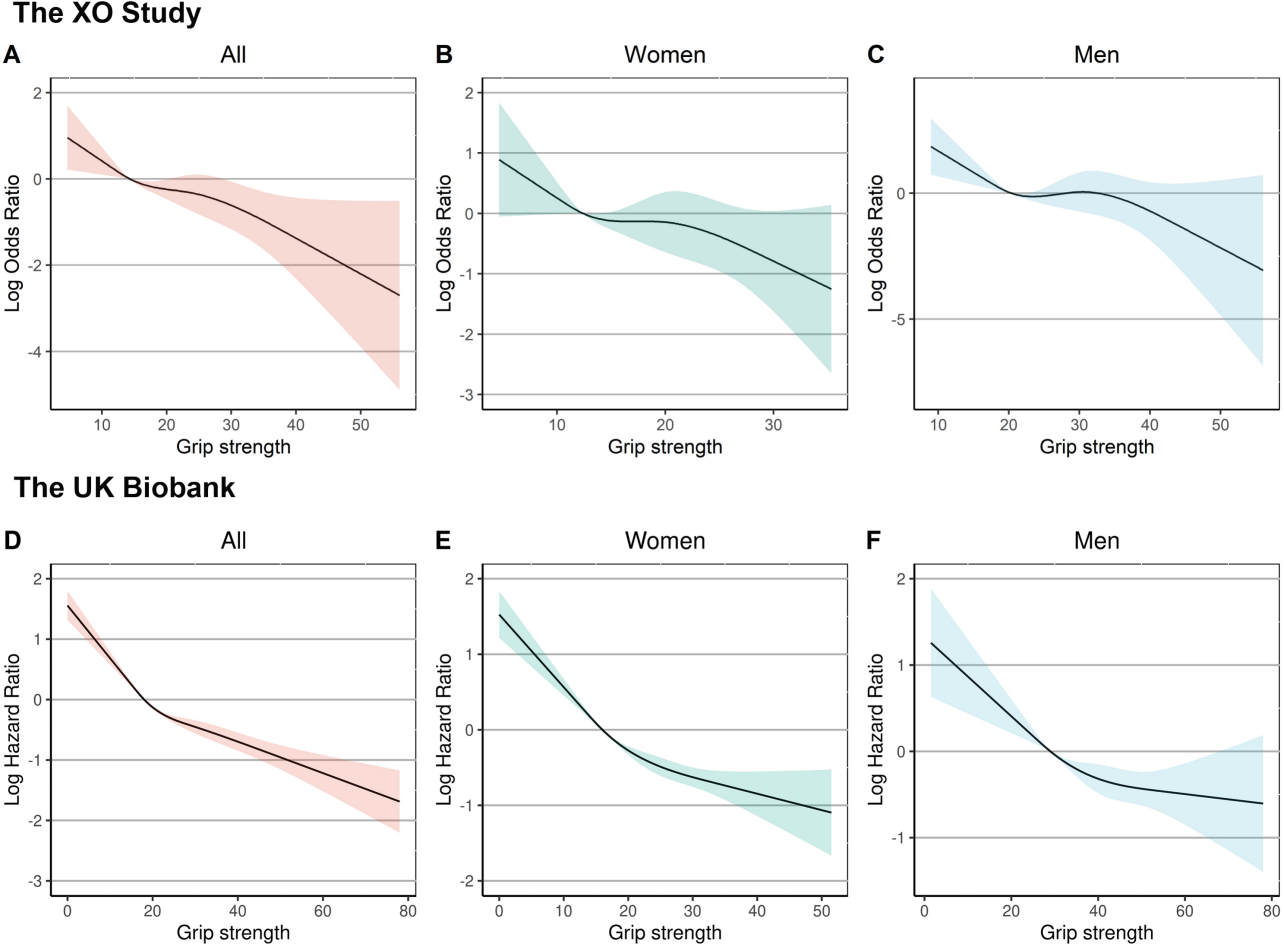
Restricted cubic spline regression models assessing the dose–response associations between grip strength and symptomatic HOA. Shading indicates 95% CI; solid lines indicate odds ratios. Adjusted log odds ratios for incident symptomatic HOA per grip strength increments are respectively estimated in all participants (A), in women (B) and in men (C) in the Xiangya Osteoarthritis (XO) Study, and in all participants (D), in women (E) and in men (F) in the UK Biobank.

A total of 2869 participants with 5461 hands were included in the final analyses (Figure 2A). Of them, 1603 (55.9%) were women, and the mean age was 63.2 years (Table S1). A total of 703 participants (968 hands) were diagnosed with radiographic HOA, and 315 participants (458 hands) reported hand pain at baseline. As shown in Table S1, hands in the lowest age‐ and sex‐specific quartile of grip strength had a slightly higher prevalence history of hand injury. Missingness was minimal, with 1.94% for BMI, 0.66% for education level and 0.84% for history of hand injury. During a mean of 3.7 years of follow‐up (follow‐up rate 80.7%), 166 (3.0%) hands developed incident symptomatic HOA. Risks were lower in the second (2.4%), third (2.9%) and fourth (2.2%) quartiles compared with the first quartile of grip strength (4.6%). Multivariable‐adjusted ORs were 0.51 (95% CI, 0.31–0.84), 0.62 (95% CI, 0.39–0.99) and 0.46 (95% CI, 0.28–0.75), respectively (*p* for trend = 0.003). Similar patterns were observed in separate analyses for men and women (Table 1). All sensitivity analyses yielded consistent results (Table S2). Restricted spline regression models also demonstrated that higher grip strength was associated with a lower risk of incident symptomatic HOA (Figure 3A–C).

**TABLE 1:** Association between grip strength and incident symptomatic HOA in the XO Study.

Symptomatic HOA	Grip strength^a^				*p* for trend
	Q1 (lowest)	Q2	Q3	Q4 (highest)	
Total					
Total number of hands, *n*	1401	1349	1324	1387	
Incident case, *n* (%)	65 (4.6)	32 (2.4)	38 (2.9)	31 (2.2)	
Crude OR (95% CI)	1.00 (reference)	0.50 (0.31, 0.80)	0.61 (0.38, 0.97)	0.47 (0.29, 0.77)	< 0.001
Adjusted OR^b^ (95% CI)	1.00 (reference)	0.51 (0.31, 0.84)	0.62 (0.39, 0.99)	0.46 (0.28, 0.75)	0.003
Women					
Number of hands, *n*	802	751	734	757	
Incident case, *n* (%)	44 (5.5)	22 (2.9)	30 (4.1)	22 (2.9)	
Crude OR (95% CI)	1.00 (reference)	0.52 (0.29, 0.92)	0.73 (0.43, 1.26)	0.52 (0.28, 0.95)	0.049
Adjusted OR^c^ (95% CI)	1.00 (reference)	0.56 (0.31, 1.00)	0.73 (0.43, 1.25)	0.47 (0.26, 0.85)	< 0.001
Men					
Number of hands, *n*	599	598	590	630	
Incident case, *n* (%)	21 (3.5)	10 (1.7)	8 (1.4)	9 (1.4)	
Crude OR (95% CI)	1.00 (reference)	0.47 (0.20, 1.11)	0.38 (0.14, 0.99)	0.40 (0.17, 0.95)	0.040
Adjusted OR^c^ (95% CI)	1.00 (reference)	0.48 (0.19, 1.22)	0.36 (0.14, 0.93)	0.40 (0.16, 1.00)	0.034

Abbreviations: CI, confidence interval; HOA, hand osteoarthritis; OR, odds ratio; XO Study, Xiangya Osteoarthritis Study.

^a^ Grip strength was divided into quartiles by age (every 2 years) and sex.

^b^ Adjusted for sex, age, education, body mass index and history of hand injury.

^c^ Adjusted for age, education, body mass index and history of hand injury.

### 3.2 Association Between Grip Strength and Incident Hospital‐Diagnosed HOA in the UK Biobank

A total of 481 582 participants without baseline hospital‐diagnosed HOA in the UK Biobank were included in cohort analyses (Figure 2B). Of them, 261 249 (54.2%) were women. The mean age was 56.4 years, and the mean BMI was 27.4 kg/m^2^ (Table S3). Missingness was minimal, with 0.23% for BMI and 0.85% for education level. During a mean follow‐up of 13.3 years, 3072 (0.6%) participants developed incident hospital‐diagnosed HOA. Compared to the first age and sex‐specific quartile of grip strength, HRs for incident hospital‐diagnosed HOA were 0.70 (95% CI, 0.64–0.77), 0.61 (95% CI, 0.55–0.67) and 0.54 (95% CI, 0.48–0.59) in the second, third and fourth quartiles of grip strength, respectively (*p* for trend < 0.001). Sex‐specific analyses showed similar findings (Table 2). Restricted spline regression models also demonstrated that higher grip strength was associated with a lower risk of incident hospital‐diagnosed HOA (Figure 3D–F). Excluding participants who developed hospital‐diagnosed HOA during the first 1 or 2 years of follow‐up did not materially change the observed associations (Table 2).

**TABLE 2:** Association between grip strength and incident hospital‐diagnosed HOA in the UK Biobank.

Hospital‐diagnosed HOA	Grip strength^a^				*p* for trend
	Q1 (lowest)	Q2	Q3	Q4 (highest)	
Total					
Number of participants, *n*	128 253	120 687	119 647	112 995	
Mean follow‐up time (mean ± SD), days	4765.61 (823.10)	4846.93 (733.58)	4890.08 (707.22)	4941.53 (682.45)	
Crude HR (95% CI)	1.00 (reference)	0.69 (0.63, 0.76)	0.60 (0.55, 0.66)	0.52 (0.47, 0.58)	< 0.0001
Adjusted HR^b^ (95% CI)	1.00 (reference)	0.70 (0.64, 0.77)	0.61 (0.55, 0.67)	0.54 (0.48, 0.59)	< 0.0001
1‐year lag^c^ (95% CI)	1.00 (reference)	0.72 (0.66, 0.80)	0.63 (0.57, 0.70)	0.56 (0.51, 0.63)	< 0.0001
2‐year lag^d^ (95% CI)	1.00 (reference)	0.73 (0.66, 0.81)	0.65 (0.59, 0.72)	0.59 (0.53, 0.66)	< 0.0001
Women					
Number of participants, *n*	70 368	65 535	65 524	59 822	
Mean follow‐up time (mean ± SD), days	4820.78 (729.28)	4876.97 (677.21)	4922.41 (642.80)	4974.50 (623.86)	
Crude HR (95% CI)	1.00 (reference)	0.67 (0.60, 0.75)	0.59 (0.52, 0.66)	0.48 (0.43, 0.55)	< 0.0001
Adjusted HR^e^ (95% CI)	1.00 (reference)	0.67 (0.60, 0.75)	0.59 (0.53, 0.66)	0.49 (0.44, 0.56)	< 0.0001
1‐year lag^c^ (95% CI)	1.00 (reference)	0.69 (0.62, 0.78)	0.61 (0.54, 0.69)	0.52 (0.46, 0.59)	< 0.0001
2‐year lag^d^ (95% CI)	1.00 (reference)	0.70 (0.62, 0.79)	0.64 (0.56, 0.72)	0.55 (0.48, 0.62)	< 0.0001
Men					
Number of participants, *n*	57 885	55 152	54 123	53 173	
Mean follow‐up time (mean ± SD), days	4698.56 (919.97)	4811.23 (793.92)	4850.94 (776.31)	4904.43 (741.12)	
Crude HR (95% CI)	1.00 (reference)	0.77 (0.65, 0.91)	0.64 (0.54, 0.77)	0.63 (0.53, 0.76)	< 0.0001
Adjusted HR^e^ (95% CI)	1.00 (reference)	0.78 (0.66, 0.92)	0.65 (0.55, 0.78)	0.64 (0.53, 0.77)	< 0.0001
1‐year lag^c^ (95% CI)	1.00 (reference)	0.80 (0.67, 0.95)	0.68 (0.57, 0.82)	0.67 (0.55, 0.81)	< 0.0001
2‐year lag^d^ (95% CI)	1.00 (reference)	0.83 (0.69, 0.99)	0.69 (0.57, 0.84)	0.70 (0.58, 0.85)	0.0002

Abbreviations: CI, confidence interval; HOA, hand osteoarthritis; HR, hazard ratio.

^a^ Grip strength was divided into quartiles by age (every 2 years) and sex.

^b^ Adjusted for sex, age, education, body mass index and history of hand injury.

^c^ Analysis was performed by excluding the HOA cases that developed within 1 year after the index date and adjusted for covariates the same as multivariable‐adjusted model.

^d^ Analysis was performed by excluding the HOA cases that developed within 2 years after the index date and adjusted for covariates the same as multivariable‐adjusted model.

^e^ Adjusted for age, education, body mass index and history of hand injury.

### 3.3 Causal Association Between Grip Strength and Incident Symptomatic HOA in the XO Study

**FIGURE 4:**
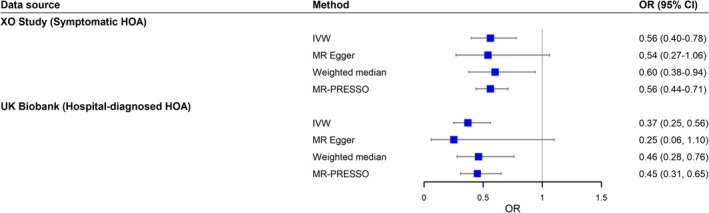
Forest plot of Mendelian randomization estimates for grip strength on symptomatic/hospital‐diagnosed HOA. CI, confidence interval; HOA, hand osteoarthritis; IVW, inverse‐variance weighted; MR, Mendelian randomization; MR‐PRESSO, MR pleiotropy residual sum and outlier; OR, odds ratio; XO Study, Xiangya Osteoarthritis Study.

As shown in Figures 4 and S1, each kg increase in grip strength corresponded to a ~40% decrease in the risk of incident symptomatic HOA (OR = 0.56, 95% CI, 0.40–0.78, *p* < 0.001). This result was consistent across different sensitivity analyses, with ORs ranging from 0.54 to 0.60 (Figure 4). No outlier was detected by the MR‐PRESSO test (*p* = 0.987). The *F*‐statistic was 26.3, indicating no evidence of weak instrument bias. Cochran's *Q* test (*p* = 0.979) indicated no heterogeneity in the IVW estimate. Table S4 provides IV for MR analysis in the XO Study.

### 3.4 Causal Association Between Grip Strength and Incident Hospital‐Diagnosed HOA in the UK Biobank

Genetically predicted grip strength was also inversely associated with the risk of incident hospital‐diagnosed HOA among the participants from the UK Biobank. For each kg increment in grip strength, the risk of hospital‐diagnosed HOA decreased by approximately 60% (OR = 0.37, 95% CI, 0.25–0.56, *p* < 0.001) (Figure 4 and Figure S2). Sensitivity analyses using robust methods also gave consistent estimates, with ORs ranging from 0.25 to 0.46 per kg increase in grip strength (Figure 4). Table S5 details the characteristics of IVs used in MR analysis in the UK Biobank.

## 4 Discussion

### 4.1 Statement of Principal Findings

Our studies performed in two large, diverse cohorts from China and the United Kingdom consistently suggested that higher grip strength is associated with a lower risk of incident symptomatic HOA. Furthermore, MR analyses performed in these cohorts indicated that genetically predicted higher grip strength was associated with a lower risk of symptomatic HOA, suggesting that the study findings are unlikely to be substantially affected by residual confounding or reverse causation.

### 4.2 Comparison With Previous Studies

To the best of our knowledge, only one longitudinal study has previously evaluated the association between grip strength and incident radiographic HOA, revealing a positive association in both men and women [15]. However, the study did not consider symptomatic HOA, a more clinically relevant disease phenotype. Moreover, approximately 70% of the participants were lost to follow‐up, which may potentially introduce selection bias. Another two cohort studies investigated the association of grip strength with the progression of HOA [16, 17]. One study found that higher grip strength was associated with a higher risk of progression of radiographic HOA in men [16], whereas another failed to confirm an association [17]. Notably, both studies were conducted among the HOA population, and baseline grip strength may be affected by HOA presence, potentially inducing collider bias and eliciting null or paradoxical findings [18]. In contrast, our study leveraged data from two substantially larger cohorts (the XO Study, *n* = 2869; the UK Biobank, *n* = 481 582). The XO Study has a relatively high follow‐up rate (80.7%). Furthermore, we applied MR analysis, a design conceptualized as a natural experiment, impervious to residual confounding and reverse causation effects [34]. The results from both studies showed consistent findings that higher grip strength reduces the risk of incident symptomatic HOA.

In addition, one previous cross‐sectional study has reported an association between lower grip strength and a higher prevalence of symptomatic HOA [35], a finding consistent with ours. However, given that symptomatic HOA is associated with functional limitations and disability, which may potentially impair grip strength performance [19], resulting in a reverse causal association between lower grip strength and higher prevalence of symptomatic HOA [12]. Our longitudinal study is the first to unveil a significant association between higher grip strength and a lower risk of incident symptomatic HOA. This finding was further strengthened by similar findings in an independent cohort. Moreover, we performed MR analyses in both cohorts, providing additional evidence to support the causal inference for this association.

### 4.3 Potential Mechanisms

Recent studies have discovered that higher grip strength can attenuate the load on the hand joints by altering their alignment [36]. This adjustment reduces the strain on damaged joint structures, lowering the potential for inflammation and consequent pain [36]. Furthermore, it is worth noting that systemic processes, such as inflammation, may play a more significant role in the development and progression of HOA [37]. There is also a strong possibility that grip strength enhances an anti‐nociceptive mechanism by modulating endogenous analgesia [38] and/or by exerting anti‐inflammatory effects that can reduce peripheral and central sensitization [39]. As a result, individuals with better grip strength may have better function and less pain [40]. In contrast, weaker grip strength has been found to predispose to periarticular conditions such as tenosynovitis or enthesopathy, which are other causes of hand pain [11].

### 4.4 Strengths and Limitations

There are several strengths to our study. Firstly, the demographic heterogeneity of our cohorts, encompassing the Chinese and the UK populations, strengthens the observational study's validity and broadens its generalizability. Secondly, the utilization of MR analysis not only reinforces the results from our cohort study but also provides the primary evidence for causal associations between grip strength and symptomatic HOA. Finally, the robustness of our analysis was underscored by multiple sensitivity analyses, thereby enhancing the reliability of our conclusions.

Nevertheless, our study has some limitations. First, the hand‐specific symptomatic OA was not assessed in the UK Biobank cohort, necessitating the use of average grip strength as an exposure to investigate its association with incident clinically diagnosed symptomatic HOA. However, the potential non‐differential misclassification of grip strength may bias the association towards the null. Secondly, reverse causation could complicate the interpretation of our results, as early or subclinical symptoms of HOA might have influenced grip strength before the diagnosis. Nevertheless, our sensitivity analyses, including exclusion of participants with radiographic HOA or hand pain at baseline, or adjusted for these conditions in the XO study, and incorporated 1‐year and 2‐year lag‐time analyses in the UK Biobank, yielded results consistent with our primary findings, supporting the robustness and validity of our conclusions. Thirdly, owing to the substantial ethnic differences between populations in the XO Study and the UK Biobank, which complicate two‐sample MR analyses, we undertook two separate one‐sample MR analyses. Nevertheless, the consistent results supported the robustness of our findings across populations. Fourthly, despite employing various robust MR methods and obtaining consistent estimates, we cannot exclude horizontal pleiotropy as it is not empirically verifiable. Finally, our findings warrant further validation in other populations, as the relatively low prevalence of symptomatic HOA in the UK Biobank may limit the generalizability of our results.

### 4.5 Implications for Clinicians and Policymakers

Given the significant burden of symptomatic HOA, there is an urgent call for understanding the risk factors for this disease to guide the development of preventive strategies, particularly low‐cost interventions targeting modifiable risk factors. Our findings suggest that enhancing grip strength through targeted exercise can effectively alleviate the burden of symptomatic HOA by preventing its occurrence. The clinical utility of grip strength assessment, owing to its ease and non‐invasive nature, could facilitate early identification of individuals at elevated risk for symptomatic HOA. Furthermore, recent guidelines recommend grip‐strengthening exercise as a core treatment for relieving pain in people with HOA [41, 42]. Our study offers empirical support for the integration of specific grip‐strengthening exercises into HOA management guidelines. These exercises, involving activities like ball or tube squeezing, elastic band use and wrist rolls, could be prominent features in public recommendations for physical activity and health worldwide, serving as self‐management targets for individuals experiencing hand pain. Variability in the response to different exercise types and regimens on symptomatic HOA necessitates additional research to develop individualized, optimized exercise programmes for affected patients [40]. Such efforts will enable the customization of interventions to individual requirements, ultimately leading to enhanced management and prevention of symptomatic HOA.

In summary, higher grip strength was associated with a lower risk of incident symptomatic HOA. These findings offer empirical evidence that enhancing grip strength through targeted exercise may help reduce the individual and societal burden of symptomatic HOA by preventing its occurrence.

## Funding

This work was supported by the National Key Research and Development Program (2022YFC3601900 and 2022YFC2505500), the National Natural Science Foundation of China (81 930 071, 82 072 502, U21A20352, 82 372 474 and 82 302 771), the Natural Science Foundation of Hunan Province (2022JJ40821), the Scientific Research Program of the Education Department of Hunan Province (296840) and the Scientific Research Program of FuRong Laboratory (2023SK2100, 2024PT5108 and 2025PT5001).

## Ethics Statement

The Xiangya Osteoarthritis Study has been approved by the Research Ethical Committee of Xiangya Hospital, Central South University (201510506), and all participants gave informed written consent for their participation in the studies. Ethics approval for the UK Biobank was obtained from the North West Centre for Research Ethics Committee (11/NW/0382), and informed consent was obtained from all participants.

## Conflicts of Interest

All authors have completed the ICMJE uniform disclosure form at http://www.icmje.org/disclosure‐of‐interest/and declare support from the National Natural Science Foundation of China, the National Key Research and Development Program and the Natural Science Foundation of Hunan Province for the submitted work; no financial relationships with any organizations that might have an interest in the submitted work in the previous 3 years; no other relationships or activities that could appear to have influenced the submitted work.

## Supporting information


**Table S1:** Baseline characteristics of participants included in the XO Study.
**Table S2:** Sensitivity analyses of association between grip strength and incident symptomatic HOA in the XO Study.
**Table S3:** Baseline characteristics of participants included in the UK Biobank.
**Table S4:** Characteristics of SNPs used in MR analysis of the effects of grip strength in incident symptomatic HOA in the XO Study.
**Table S5:** Characteristics of SNPs used in MR analysis of the effects of grip strength in incident hospital‐diagnosed HOA in the UK Biobank.
**Figure S1:** MR scatterplots of the effects of grip strength in incident symptomatic HOA in the XO Study.
**Figure S2:** MR scatterplots of the effects of grip strength in incident hospital‐diagnosed HOA in the UK Biobank.
