# Supplementary material for: Higher Grip Strength Is Associated With Reduced Risk of Incident Symptomatic Hand Osteoarthritis: Data From Two Cohort Studies
Source: J Cachexia Sarcopenia Muscle. 2026 Mar 31;17(2):e70265. doi: 10.1002/jcsm.70265 (PMC13054672; doi:10.1002/jcsm.70265)
Supplement: Supplementary file 1 — Table S1: Baseline characteristics of participants included in the XO Study. Table S2: Sensitivity analyses of association between grip strength and incident symptomatic HOA in the XO Study. Table S3: Baseline characteristics of participants included in the UK Biobank. Table S4: Characteristics of SNPs used in MR analysis of the effects of grip strength in incident symptomatic HOA in the XO Study. Table S5: Characteristics of SNPs used in MR analysis of the effects of grip strength in incident hospital‐diagnosed HOA in the UK Biobank. Figure S1: MR scatterplots of the effects of grip strength in incident symptomatic HOA in the XO Study. Figure S2: MR scatterplots of the effects of grip strength in incident hospital‐diagnosed HOA in the UK Biobank. [file JCSM-17-e70265-s001.docx]

**Supplemental Online Content**

**Higher grip strength is associated with reduced risk of incident symptomatic hand osteoarthritis:** **data from two cohort studies**

[Table S1. Baseline characteristics of participants included in the XO Study 2](#_Toc225412024)

[Table S2. Sensitivity analyses of association between grip strength and incident symptomatic HOA in the XO Study 4](#_Toc225412025)

[Table S3. Baseline characteristics of participants included in the UK Biobank 5](#_Toc225412026)

[Table S4. Characteristics of SNPs used in MR analysis of the effects of grip strength in incident symptomatic HOA in the XO Study 7](#_Toc225412027)

[Table S5. Characteristics of SNPs used in MR analysis of the effects of grip strength in incident hospital-diagnosed HOA in the UK Biobank 9](#_Toc225412028)

[Figure S1. MR scatterplots of the effects of grip strength in incident symptomatic HOA in the XO Study. 15](#_Toc225412029)

[Figure S2. MR scatterplots of the effects of grip strength in incident hospital-diagnosed HOA in the UK Biobank. 16](#_Toc225412030)

[eMethods 17](#_Toc225412031)

[REFERENCE 21](#_Toc225412032)

# Table S1. Baseline characteristics of participants included in the XO Study

|  | All in person level  (participants, n=2,869) | Quartiles of grip strength^a^ in hand level | | | | | | |
| --- | --- | --- | --- | --- | --- | --- | --- | --- |
|  |  | Q1  (hands, n=1,401) | | Q2  (hands, n=1,349) | | Q3  (hands, n=1,324) | | Q4  (hands, n=1,387) |
| **Total** |  |  | |  | |  | |  |
| Sex (women), n (%) | 1,603 (55.9) | 802 (57.2) | | 751 (55.7) | | 734 (55.4) | | 757 (54.6) |
| Age (mean ± SD), years | 63.2 ± 8.7 | 62.9 ± 8.6 | | 63.1 ± 8.7 | | 63.2 ± 8.7 | | 63.1 ± 8.8 |
| BMI, n (%) |  |  | |  | |  | |  |
| Underweight | 79 (2.8) | 56 (4.0) | | 35 (2.6) | | 30 (2.3) | | 24 (1.8) |
| Normal | 1,434 (51.0) | 788 (56.6) | | 714 (54.0) | | 652 (50.7) | | 577 (42.6) |
| Overweight | 968 (34.4) | 395 (28.4) | | 443 (33.5) | | 467 (36.3) | | 547 (40.4) |
| Obese | 333 (11.8) | 153 (11.0) | | 131 (9.9) | | 137 (10.7) | | 206 (15.2) |
| Education (educated, %)^b^ | 1,983 (69.6) | 955 (68.8) | | 909 (67.5) | | 938 (71.3) | | 991 (72.2) |
| Hand injury history, n (%) | 92 (3.2) | 56 (4.0) | | 46 (3.4) | | 27 (2.1) | | 39 (2.8) |
| **Men** |  |  | |  | |  | |  |
| Number of participants/hands, n | 1,266 | 599 | | 598 | | 590 | | 630 |
| Age (mean ± SD), years | 64.0 ± 8.5 | 63.5 ± 8.3 | | 64.0 ± 8.5 | | 64.0 ± 8.7 | | 64.0 ± 8.6 |
| BMI, n (%) |  |  | |  | |  | |  |
| Underweight | 40 (3.2) | 35 (5.9) | | 18 (3.1) | | 12 (2.1) | | 9 (1.5) |
| Normal | 724 (58.5) | 387 (65.0) | | 350 (59.5) | | 327 (57.4) | | 317 (51.9) |
| Overweight | 372 (30.1) | 130 (21.9) | | 181 (30.8) | | 183 (32.1) | | 220 (36.0) |
| Obese | 102 (8.2) | 43 (7.2) | | 39 (6.6) | | 48 (8.4) | | 65 (10.6) |
| Education (educated, %)^b^ | 1,105 (87.9) | 513 (86.5) | | 513 (85.8) | | 524 (89.3) | | 561 (90.0) |
| Hand injury history, n (%) | 56 (4.5) | 31 (5.2) | 23 (3.8) | | 21 (3.6) | | 25 (4.0) | |
| **Women** |  |  |  | |  | |  | |
| Number of participants/hands, n | 1,603 | 802 | 751 | | 734 | | 757 | |
| Age (mean ± SD), years | 62.5 ± 8.8 | 62.4 ± 8.8 | 62.4 ± 8.8 | | 62.5 ± 8.7 | | 62.3 ± 8.8 | |
| BMI, n (%) |  |  |  | |  | |  | |
| Underweight | 39 (2.5) | 21 (2.6) | 17 (2.3) | | 18 (2.5) | | 15 (2.0) | |
| Normal | 710 (45.0) | 401 (50.3) | 364 (49.5) | | 325 (45.4) | | 260 (35.0) | |
| Overweight | 596 (37.8) | 265 (33.3) | 262 (35.7) | | 284 (39.7) | | 327 (44.0) | |
| Obese | 231 (14.7) | 110 (13.8) | 92 (12.5) | | 89 (12.4) | | 141 (19.0) | |
| Education (educated, %)^b^ | 878 (55.1) | 442 (55.5) | 396 (52.9) | | 414 (56.8) | | 430 (57.3) | |
| Hand injury history, n (%) | 36 (2.3) | 25 (3.1) | 23 (3.1) | | 6 (0.8) | | 14 (1.9) | |

XO Study, Xiangya Osteoarthritis Study; HOA, hand osteoarthritis; BMI, body mass index; SD, standard deviation.

^a^ Grip strength was divided into quartiles by sex-age-specific.

^b^ Educated was defined as primary school or above.

# Table S2. Sensitivity analyses of association between grip strength and incident symptomatic HOA in the XO Study

| Symptomatic HOA | Grip strength^a^ | | |  | *P* for trend |
| --- | --- | --- | --- | --- | --- |
|  | Q1 (lowest) | Q2 | Q3 | Q4 (highest) |  |
| Person-based analysis |  |  |  |  |  |
| Total number of persons, n | 698 | 682 | 671 | 684 |  |
| Incident case, n (%) | 41 (5.9) | 18 (2·6) | 19 (2·8) | 26 (3·8) |  |
| Crude OR (95%CI) | 1.00 (reference) | 0.43 (0.25, 0.76) | 0.47 (0.27, 0.81) | 0.63 (0.38, 1.05) | < 0.001 |
| Adjusted OR^b^ (95%CI) | 1.00 (reference) | 0.42 (0.24, 0.74) | 0.46 (0.26, 0.81) | 0.59 (0.35, 0.99) | 0.020 |
| Excluding hands with only radiographic HOA or only pain at baseline | | | | | |
| Total number of hands, n | 1,035 | 1,008 | 979 | 1,013 |  |
| Incident case, n (%) | 21 (2.0) | 12 (1.2) | 8 (0.8) | 8 (0.8) |  |
| Crude OR (95%CI) | 1.00 (reference) | 0.58 (0.26, 1.31) | 0.40 (0.17, 0.94) | 0.38 (0.15, 0.97) | 0.001 |
| Adjusted OR^c^ (95%CI) | 1.00 (reference) | 0.60 (0.27, 1.34) | 0.40 (0.17, 0.95) | 0.36 (0.14, 0.89) | 0.014 |
| Further adjusted for radiographic HOA and hand pain only at baseline | | | | | |
| Total number of hands, n | 1,401 | 1,349 | 1,324 | 1,387 |  |
| Incident case, n (%) | 65 (4.6) | 32 (2.4) | 38 (2.9) | 31 (2.2) |  |
| Crude OR (95%CI) | 1.00 (reference) | 0.50 (0.31, 0.80) | 0.61 (0.38, 0.97) | 0.47 (0.29, 0.77) | < 0.001 |
| Adjusted OR^d^ (95%CI) | 1.00 (reference) | 0.50 (0.30, 0.83) | 0.65 (0.40, 1.06) | 0.48 (0.28, 0.80) | 0.007 |

XO Study, Xiangya Osteoarthritis Study; HOA, hand osteoarthritis; OR, odds ratio; CI, confidence interval.

^a^ Grip strength was divided into quartiles by age (every two years) and sex.

^b^ Adjusted for sex, age, education, body mass index and history of hand injury.

^c^ Adjusted for sex, age, education and body mass index.

^d^ Adjusted for sex, age, education, body mass index, history of hand injury, radiographic HOA and hand pain only.

# Table S3. Baseline characteristics of participants included in the UK Biobank

|  | **Grip strength^a^** | | | |  |
| --- | --- | --- | --- | --- | --- |
|  | Q1 (lowest) | Q2 | Q3 | Q4 (highest) |  |
| **Total** |  |  |  |  |  |
| Participants, n | 128,253 | 120,687 | 119,647 | 112,995 |  |
| Age (mean ± SD), years | 56.40 ± 8.1 | 56.5 ± 8.1 | 56.4 ± 8.1 | 56.4 ± 8.1 |  |
| Sex (Female), n (%) | 70,368 (54.9) | 65,535 (54.3) | 65,524 (54.8) | 59,822 (52.9) |  |
| BMI, n (%) |  |  |  |  |  |
| Normal | 42,516 (33.3) | 42,326 (35.1) | 40,707 (34.1) | 34,486 (30.6) |  |
| Overweight | 51,804 (40.6) | 50,560 (42.0) | 51,809 (43.4) | 50,300 (44.6) |  |
| Obese | 33,343 (26.1) | 27,578 (22.9) | 26,985 (22.6) | 28,066 (24.9) |  |
| Education, n (%) |  |  |  |  |  |
| Non-college | 88,094 (69.3) | 79,644 (66.6) | 77,324 (65.1) | 70,941 (63.3) |  |
| College | 37,011 (29.1) | 38,774 (32.4) | 40,306 (34.0) | 40,287 (35.9) |  |
| Unknown | 1,949 (1.5) | 1,197 (1.0) | 1,078 (0.9) | 903 (0.8) |  |
| History of hand injury, n (%) | 1,877 (1.5) | 1,513 (1.3) | 1,438 (1.2) | 1,480 (1.3) |  |
| **Men** |  |  |  |  |  |
| Participants, n | 57,885 | 55,152 | 54,123 | 53,173 |  |
| Age (mean ± SD), years | 56.7 ± 8.2 | 56.6 ± 8.2 | 56.7 ± 8.3 | 56.6 ± 8.2 |  |
| BMI, n (%) |  |  |  |  |  |
| Normal | 16,265 (28.3) | 15,174 (27.6) | 13,484 (24.9) | 10,819 (20.4) |  |
| Overweight | 26,352 (45.8) | 26,861 (48.8) | 27,504 (50.9) | 27,920 (52.6) |  |
| Obese | 14,914 (25.9) | 13,001 (23.6) | 13,063 (24.2) | 14,353 (27.0) |  |
| Education, n (%) |  |  |  |  |  |
| Non-college | 37,675 (65.8) | 35,161 (64.3) | 34,461 (64.2) | 34,192 (64.7) |  |
| College | 18,706 (32.7) | 18,930 (34.6) | 18,742 (34.9) | 18,121 (34.3) |  |
| Unknown | 908 (1.6) | 565 (1.0) | 510 (0.9) | 502 (1.0) |  |
| History of hand injury, n (%) | 1,144 (2.0) | 955 (1.7) | 928 (1.7) | 976 (1.8) | |
| **Women** |  |  |  |  |  |
| Participants, n | 70,368 | 65,535 | 65,524 | 59,822 |  |
| Age (mean ± SD), years | 56.2 ± 8.1 | 56.4 ± 8.0 | 56.2 ± 8.0 | 56.2 ± 8.0 |  |
| BMI n (%) |  |  |  |  |  |
| Normal | 26,251 (37.4) | 27,152 (41.5) | 27,223 (41.6) | 23,667 (39.6) |  |
| Overweight | 25,452 (36.3) | 23,699 (36.2) | 24,305 (37.1) | 22,380 (37.4) |  |
| Obese | 18,429 (26.3) | 14,577 (22.3) | 13,922 (21.3) | 13,713 (22.9) |  |
| Education, n (%) |  |  |  |  |  |
| Non-college | 50,419 (72.3) | 44,483 (68.5) | 42,863 (65.9) | 36,749 (62.0) |  |
| College | 18,305 (26.2) | 19,844 (30.5) | 21,564 (33.2) | 22,166 (37.4) |  |
| Unknown | 1,041 (1.5) | 632 (1.0) | 568 (0.9) | 401 (0.7) |  |
| History of hand injury, n (%) | 733 (1.0) | 558 (0.9) | 510 (0.8) | 504 (0.8) |  |

HOA, hand osteoarthritis; BMI, body mass index; SD, standard deviation.

^a^ Grip strength was divided into quartiles by age- and sex-specific.

# Table S4. Characteristics of SNPs used in MR analysis of the effects of grip strength in incident symptomatic HOA in the XO Study

| Targe SNPs | Effect allele | Association with grip strength | | |  | Association with symptomatic HOA | | |
| --- | --- | --- | --- | --- | --- | --- | --- | --- |
|  |  | beta | SE | P value |  | beta | SE | P value |
| rs10237543 | A | -0.104088 | 0.0219288 | 2.07E-06 |  | 0.127722 | 0.0875844 | 0.1447662 |
| rs1048723 | G | -0.103648 | 0.0232729 | 8.44E-06 |  | 0.035212 | 0.0933822 | 0.706119 |
| rs11020732 | C | -0.232853 | 0.0454064 | 2.93E-07 |  | 0.231966 | 0.179089 | 0.1952321 |
| rs11582421 | G | -0.120521 | 0.02708 | 8.57E-06 |  | 0.0313242 | 0.108642 | 0.7730987 |
| rs11783501 | C | -0.106067 | 0.0229635 | 3.86E-06 |  | 0.0300866 | 0.0919122 | 0.7434103 |
| rs11858758 | A | 0.167963 | 0.0339477 | 7.51E-07 |  | -0.22102 | 0.136693 | 0.1058991 |
| rs138602916 | A | 0.302387 | 0.0676969 | 7.94E-06 |  | -0.546226 | 0.39239 | 0.08195392 |
| rs139276082 | A | -0.531445 | 0.112318 | 2.23E-06 |  | -0.0783673 | 0.467184 | 0.8667846 |
| rs146341697 | C | -0.640395 | 0.118224 | 6.07E-08 |  | 0.420928 | 0.47639 | 0.3769237 |
| rs148112920 | G | 0.401887 | 0.0888195 | 6.05E-06 |  | -0.210269 | 0.357967 | 0.5569365 |
| rs148436349 | G | -0.22411 | 0.0497075 | 6.53E-06 |  | -0.139266 | 0.199934 | 0.486079 |
| rs148817853 | A | -0.35739 | 0.0808051 | 9.74E-06 |  | 0.42658 | 0.326965 | 0.1920071 |
| rs187013616 | A | -0.540645 | 0.118817 | 5.36E-06 |  | 0.783488 | 0.473913 | 0.04914169 |
| rs193042145 | C | -0.279283 | 0.0631009 | 9.60E-06 |  | 0.102736 | 0.249366 | 0.6803462 |
| rs201790888 | C | -0.318678 | 0.0703848 | 5.96E-06 |  | 0.446532 | 0.281192 | 0.1122864 |
| rs234173 | A | -0.551749 | 0.118346 | 3.13E-06 |  | -0.331298 | 0.459402 | 0.4708175 |
| rs2519811 | G | -0.107186 | 0.0224974 | 1.89E-06 |  | 0.055416 | 0.0901781 | 0.5388733 |
| rs371121375 | G | -0.764992 | 0.171317 | 7.99E-06 |  | 0.738348 | 0.619101 | 0.2330212 |
| rs55954399 | T | -0.405489 | 0.0910497 | 8.45E-06 |  | -0.0932431 | 0.368637 | 0.8003142 |
| rs60070153 | A | 0.113897 | 0.0250505 | 5.45E-06 |  | -0.0381917 | 0.099938 | 0.7023468 |
| rs62525335 | G | -0.167713 | 0.034721 | 1.36E-06 |  | 0.145125 | 0.138066 | 0.2931999 |
| rs76590312 | C | 0.143207 | 0.0318185 | 6.77E-06 |  | -0.250542 | 0.159253 | 0.05783267 |
| rs78871815 | A | -0.194304 | 0.0421257 | 3.98E-06 |  | 0.0623683 | 0.168026 | 0.7105013 |
| rs78963381 | T | -0.198751 | 0.0427397 | 3.32E-06 |  | 0.131789 | 0.169494 | 0.4368384 |
| rs892836 | G | -0.104596 | 0.0233899 | 7.76E-06 |  | 0.00751448 | 0.0933649 | 0.9358515 |
| rs9997523 | G | -0.124956 | 0.0274577 | 5.34E-06 |  | 0.0158143 | 0.108911 | 0.8845503 |

SNP, single nucleotide polymorphism; MR, Mendelian randomization; HOA, hand osteoarthritis; XO Study, Xiangya Osteoarthritis Study; SE, standard error.

# Table S5. Characteristics of SNPs used in MR analysis of the effects of grip strength in incident hospital-diagnosed HOA in the UK Biobank

| Targe SNPs | Effect allele | Association with grip strength | | |  | Association with symptomatic HOA | | |
| --- | --- | --- | --- | --- | --- | --- | --- | --- |
|  |  | beta | SE | P value |  | beta | SE | P value |
| rs10097534 | G | -0.0131645 | 0.00192735 | 8.47E-12 |  | 0.0454489 | 0.0309709 | 0.07112372 |
| rs10146501 | C | -0.00937432 | 0.00160253 | 4.92E-09 |  | -0.0266711 | 0.0211179 | 0.2066035 |
| rs1044299 | T | 0.0140532 | 0.00146622 | 9.28E-22 |  | 0.00643217 | 0.0193003 | 0.7389322 |
| rs10483727 | C | -0.00938838 | 0.00149174 | 3.10E-10 |  | -0.00204102 | 0.0196332 | 0.9172031 |
| rs10821939 | A | -0.00917443 | 0.00147416 | 4.86E-10 |  | 0.00306045 | 0.0194186 | 0.8747691 |
| rs10914452 | A | 0.00979321 | 0.00147071 | 2.76E-11 |  | 0.045456 | 0.0219411 | 0.01914549 |
| rs11002318 | A | -0.00971538 | 0.00153739 | 2.63E-10 |  | 0.0346755 | 0.0252862 | 0.08513771 |
| rs111927135 | T | 0.0139574 | 0.0018176 | 1.60E-14 |  | 0.0276857 | 0.0239446 | 0.2475838 |
| rs11204664 | C | -0.00898802 | 0.00146959 | 9.60E-10 |  | 0.00247807 | 0.0193349 | 0.8980178 |
| rs11243202 | C | 0.0119557 | 0.00146964 | 4.12E-16 |  | -0.0140544 | 0.0193411 | 0.4674367 |
| rs115484560 | G | -0.0207277 | 0.0031524 | 4.86E-11 |  | 0.0519569 | 0.041156 | 0.2067905 |
| rs11667949 | C | 0.0121331 | 0.00164151 | 1.45E-13 |  | 0.0243396 | 0.0215754 | 0.25927 |
| rs116922558 | G | -0.0242591 | 0.00383965 | 2.65E-10 |  | -0.0214576 | 0.0504989 | 0.6709 |
| rs11906450 | G | 0.0109347 | 0.00168514 | 8.65E-11 |  | 0.00801264 | 0.0221983 | 0.7181314 |
| rs11981566 | G | -0.011213 | 0.00176951 | 2.35E-10 |  | 0.0143583 | 0.0232589 | 0.5370213 |
| rs12137291 | T | 0.0136906 | 0.00153781 | 5.45E-19 |  | -0.0155266 | 0.0202527 | 0.4432929 |
| rs12142043 | A | -0.0165823 | 0.00221145 | 6.46E-14 |  | 0.0206313 | 0.0291277 | 0.4787563 |
| rs12332392 | G | -0.0097576 | 0.00148517 | 5.03E-11 |  | 0.0676765 | 0.0205853 | 0.000505212 |
| rs12375999 | T | 0.0131014 | 0.00198538 | 4.14E-11 |  | -0.0439815 | 0.0335769 | 0.09511929 |
| rs12412806 | A | -0.00938728 | 0.0016038 | 4.82E-09 |  | -0.00761457 | 0.0211192 | 0.7184349 |
| rs12414407 | C | 0.00983951 | 0.00151284 | 7.82E-11 |  | -0.0249915 | 0.0199018 | 0.20921 |
| rs12523793 | A | -0.00996106 | 0.00154903 | 1.27E-10 |  | 0.0166308 | 0.0203672 | 0.4141868 |
| rs12533489 | C | 0.0135987 | 0.00202182 | 1.74E-11 |  | 0.0255899 | 0.0266376 | 0.3367195 |
| rs12671188 | T | 0.00974873 | 0.00146261 | 2.64E-11 |  | 0.0105106 | 0.0192783 | 0.5856145 |
| rs12889267 | G | -0.0138902 | 0.00193861 | 7.78E-13 |  | 0.0297474 | 0.0255805 | 0.2448736 |
| rs12914702 | A | 0.0111248 | 0.00169881 | 5.81E-11 |  | -0.0274841 | 0.0224267 | 0.2203843 |
| rs12942267 | T | -0.00910877 | 0.0015179 | 1.96E-09 |  | -0.00399057 | 0.0199899 | 0.8417703 |
| rs13106087 | C | 0.0119986 | 0.00193349 | 5.45E-10 |  | -0.01203 | 0.0254384 | 0.6362778 |
| rs13107325 | T | -0.0266581 | 0.00276059 | 4.61E-22 |  | 0.0905647 | 0.0398541 | 0.01153103 |
| rs13150083 | A | -0.0117387 | 0.00167573 | 2.47E-12 |  | 0.0270809 | 0.0220718 | 0.2198413 |
| rs13162125 | G | -0.00984581 | 0.00163797 | 1.84E-09 |  | 0.00641485 | 0.0215774 | 0.7662413 |
| rs13227429 | C | -0.00896517 | 0.00146884 | 1.04E-09 |  | -0.00405927 | 0.0193208 | 0.8335908 |
| rs1407339 | T | 0.00901677 | 0.00149881 | 1.79E-09 |  | -0.0558759 | 0.0216621 | 0.004948205 |
| rs143384 | G | 0.023352 | 0.0014797 | 4.16E-56 |  | -0.0344673 | 0.02425 | 0.07761032 |
| rs1434095 | C | 0.0132498 | 0.00221826 | 2.33E-09 |  | -0.0220814 | 0.0292464 | 0.4502417 |
| rs150330307 | C | -0.0325322 | 0.00412313 | 3.02E-15 |  | -0.0114349 | 0.0543681 | 0.8334147 |
| rs1503738 | G | -0.00911969 | 0.0015384 | 3.07E-09 |  | 0.0135449 | 0.0202302 | 0.5031515 |
| rs153754 | G | -0.00921443 | 0.00152656 | 1.58E-09 |  | -0.00459461 | 0.0201069 | 0.8192507 |
| rs1703599 | A | -0.0093497 | 0.00153576 | 1.14E-09 |  | -0.000904008 | 0.0202172 | 0.9643346 |
| rs17282763 | C | 0.00989981 | 0.00160131 | 6.32E-10 |  | -0.0590873 | 0.023214 | 0.005458676 |
| rs17597864 | C | -0.0119041 | 0.00184682 | 1.15E-10 |  | 0.00865013 | 0.024382 | 0.7227584 |
| rs17630248 | C | -0.00970657 | 0.00153427 | 2.51E-10 |  | -0.0163108 | 0.0202241 | 0.4199539 |
| rs1812395 | G | 0.0105599 | 0.0015186 | 3.56E-12 |  | -0.0100927 | 0.0200097 | 0.6139868 |
| rs188499496 | A | 0.0103554 | 0.0015121 | 7.47E-12 |  | -0.0254634 | 0.0199207 | 0.2011658 |
| rs193536 | T | 0.00979179 | 0.00157426 | 4.97E-10 |  | 0.00673785 | 0.0207959 | 0.7459393 |
| rs1981612 | A | 0.00900183 | 0.00150471 | 2.20E-09 |  | -0.0285606 | 0.0197949 | 0.149069 |
| rs1985613 | C | 0.0091473 | 0.00156234 | 4.77E-09 |  | 0.00409024 | 0.0205724 | 0.8424027 |
| rs2075060 | A | 0.0148713 | 0.00146899 | 4.35E-24 |  | -0.00329667 | 0.0193126 | 0.864459 |
| rs2131371 | C | 0.0104688 | 0.00158858 | 4.40E-11 |  | 0.0232642 | 0.0208716 | 0.2650067 |
| rs217181 | T | 0.0117979 | 0.00183937 | 1.42E-10 |  | 0.00222933 | 0.0242178 | 0.9266555 |
| rs2194411 | A | 0.0147036 | 0.00222241 | 3.69E-11 |  | -0.0311043 | 0.029224 | 0.2871733 |
| rs2194747 | G | 0.0112532 | 0.0016138 | 3.10E-12 |  | -0.0346339 | 0.0212113 | 0.1025096 |
| rs2303083 | A | -0.0120185 | 0.00183951 | 6.42E-11 |  | -0.0268942 | 0.0241826 | 0.2660831 |
| rs2457533 | G | -0.0160031 | 0.00193239 | 1.22E-16 |  | 0.0488867 | 0.0300499 | 0.05188428 |
| rs2532111 | G | 0.0110903 | 0.00153086 | 4.34E-13 |  | 0.0377352 | 0.0245217 | 0.06192056 |
| rs2564924 | T | -0.0109189 | 0.00146303 | 8.44E-14 |  | 0.0252225 | 0.0192373 | 0.1898149 |
| rs2631360 | A | -0.0114713 | 0.00145412 | 3.05E-15 |  | 0.0691022 | 0.0202356 | 0.000319054 |
| rs2815731 | A | -0.0092811 | 0.00154191 | 1.75E-09 |  | 0.000822249 | 0.0202912 | 0.9676767 |
| rs2871865 | G | -0.0224816 | 0.00226911 | 3.85E-23 |  | 0.0416844 | 0.0299619 | 0.1641517 |
| rs2871960 | C | 0.0124405 | 0.0014624 | 1.79E-17 |  | -0.0454997 | 0.0217682 | 0.01830037 |
| rs2891409 | T | 0.0164265 | 0.00167549 | 1.08E-22 |  | 0.0398798 | 0.027358 | 0.07246112 |
| rs2974395 | A | 0.0102499 | 0.0016185 | 2.41E-10 |  | -0.0118056 | 0.021326 | 0.5798683 |
| rs3118914 | T | -0.0179122 | 0.0017679 | 3.99E-24 |  | 0.0276946 | 0.0232496 | 0.2335789 |
| rs34030812 | C | -0.00958195 | 0.00150763 | 2.08E-10 |  | 0.0134456 | 0.0198743 | 0.498704 |
| rs34587452 | C | -0.0111638 | 0.00176891 | 2.77E-10 |  | 0.000922385 | 0.0232863 | 0.9684036 |
| rs34588175 | A | -0.0210966 | 0.00198975 | 2.90E-26 |  | 0.0239735 | 0.026195 | 0.3600909 |
| rs34845616 | A | 0.010745 | 0.00169709 | 2.43E-10 |  | -0.00385763 | 0.0223838 | 0.86317 |
| rs35021449 | T | -0.0188904 | 0.00283339 | 2.61E-11 |  | -0.00451742 | 0.0377936 | 0.9048565 |
| rs35139284 | T | -0.0144344 | 0.00156508 | 2.89E-20 |  | 0.0209039 | 0.020677 | 0.3120287 |
| rs35467921 | T | 0.00906121 | 0.0014864 | 1.09E-09 |  | 0.00417767 | 0.0195568 | 0.8308455 |
| rs35609019 | C | 0.00961542 | 0.00152999 | 3.29E-10 |  | 0.0374234 | 0.0244592 | 0.06300451 |
| rs35701422 | C | -0.00943586 | 0.00150069 | 3.22E-10 |  | -0.0212018 | 0.0197497 | 0.2830367 |
| rs374532236 | T | 0.0183557 | 0.00151301 | 7.16E-34 |  | -0.0200893 | 0.0199376 | 0.3136418 |
| rs3771498 | T | 0.0140902 | 0.00145631 | 3.84E-22 |  | -0.0694329 | 0.0202386 | 0.000300987 |
| rs3788649 | C | 0.0102139 | 0.00165209 | 6.31E-10 |  | -0.0545205 | 0.0244918 | 0.01300463 |
| rs4073729 | A | -0.0120709 | 0.0020231 | 2.42E-09 |  | 0.0118461 | 0.0266982 | 0.657257 |
| rs4121165 | A | -0.0110577 | 0.00178081 | 5.32E-10 |  | -0.0094542 | 0.0234628 | 0.68699 |
| rs413130 | T | -0.0101762 | 0.00146089 | 3.27E-12 |  | 0.0032189 | 0.0191905 | 0.866792 |
| rs4252548 | T | -0.0312972 | 0.00497112 | 3.06E-10 |  | 0.147893 | 0.0709026 | 0.01849585 |
| rs4420634 | A | 0.0167786 | 0.00176579 | 2.06E-21 |  | -0.00478599 | 0.0232414 | 0.8368496 |
| rs4553566 | C | -0.0104651 | 0.00145833 | 7.17E-13 |  | 0.0972203 | 0.0196813 | 3.91E-07 |
| rs4575361 | T | -0.0113157 | 0.0015678 | 5.29E-13 |  | -0.0425375 | 0.0243684 | 0.04044031 |
| rs4586733 | C | 0.0100485 | 0.00168227 | 2.33E-09 |  | -0.0328843 | 0.0221332 | 0.137346 |
| rs4621706 | T | -0.0110283 | 0.00147988 | 9.18E-14 |  | -0.0486828 | 0.0216133 | 0.01214705 |
| rs4737446 | T | 0.0104146 | 0.00158467 | 4.96E-11 |  | -0.00831948 | 0.0208825 | 0.690339 |
| rs475390 | A | -0.0114482 | 0.00174687 | 5.62E-11 |  | -0.00460886 | 0.0230036 | 0.841204 |
| rs4784329 | C | -0.0128227 | 0.00147652 | 3.81E-18 |  | -0.021562 | 0.0194404 | 0.2673734 |
| rs4785683 | A | -0.00898393 | 0.00146352 | 8.33E-10 |  | 0.0267397 | 0.0192713 | 0.1652772 |
| rs4797782 | C | -0.00885174 | 0.00147198 | 1.82E-09 |  | -0.031184 | 0.0193408 | 0.1068866 |
| rs4865786 | G | -0.0090275 | 0.00146711 | 7.59E-10 |  | 0.0240401 | 0.0193277 | 0.2135667 |
| rs4886716 | C | 0.0107258 | 0.00168048 | 1.74E-10 |  | -0.036036 | 0.0221141 | 0.1031969 |
| rs57908212 | C | 0.00880918 | 0.00146882 | 2.00E-09 |  | -0.0333991 | 0.0242645 | 0.08433996 |
| rs585721 | C | -0.00977165 | 0.00149853 | 6.99E-11 |  | 0.0155623 | 0.0197133 | 0.4298585 |
| rs61734601 | A | -0.0285205 | 0.0026283 | 1.97E-27 |  | -0.0562651 | 0.0344989 | 0.1029065 |
| rs61818103 | T | 0.0145877 | 0.00226673 | 1.23E-10 |  | 0.00862779 | 0.0297765 | 0.7720062 |
| rs62004866 | G | -0.0197405 | 0.00236566 | 7.15E-17 |  | 0.0205996 | 0.0312279 | 0.5094756 |
| rs62055696 | G | -0.0149502 | 0.00176905 | 2.89E-17 |  | 0.0877007 | 0.0239263 | 0.000123449 |
| rs635538 | A | -0.0214077 | 0.00260654 | 2.16E-16 |  | 0.0690293 | 0.0415284 | 0.04823435 |
| rs6433478 | C | 0.00977513 | 0.00148225 | 4.26E-11 |  | 0.00514589 | 0.0195036 | 0.7919007 |
| rs649129 | T | -0.0117625 | 0.00179425 | 5.54E-11 |  | 0.00203082 | 0.0236153 | 0.9314697 |
| rs654585 | C | -0.00921174 | 0.00146254 | 3.01E-10 |  | -0.0190768 | 0.0192877 | 0.3226308 |
| rs6669277 | A | -0.016312 | 0.00189454 | 7.31E-18 |  | 0.0280778 | 0.0244721 | 0.2512412 |
| rs6882168 | T | -0.0107383 | 0.0015418 | 3.29E-12 |  | 0.0252255 | 0.020289 | 0.2137538 |
| rs6948467 | A | -0.00910472 | 0.00150144 | 1.33E-09 |  | 0.00246705 | 0.0197181 | 0.9004318 |
| rs6962338 | G | -0.0208339 | 0.00353693 | 3.85E-09 |  | -0.0727713 | 0.0470089 | 0.1216147 |
| rs6977081 | T | 0.0151542 | 0.0015628 | 3.11E-22 |  | -0.0157546 | 0.0205736 | 0.4438136 |
| rs7011006 | T | -0.00959157 | 0.00157172 | 1.04E-09 |  | 0.00368828 | 0.0206549 | 0.8582779 |
| rs7034200 | A | 0.00865111 | 0.0014547 | 2.73E-09 |  | 0.00314609 | 0.019168 | 0.8696266 |
| rs7071654 | G | 0.0133787 | 0.00198847 | 1.72E-11 |  | 0.0131006 | 0.0261404 | 0.6162551 |
| rs71298370 | A | 0.0173716 | 0.00269359 | 1.12E-10 |  | 0.00468561 | 0.0354134 | 0.8947378 |
| rs7196917 | G | -0.0114499 | 0.00146913 | 6.51E-15 |  | 0.0156686 | 0.0193622 | 0.4183799 |
| rs721101 | C | 0.00957229 | 0.0016355 | 4.83E-09 |  | 0.000245532 | 0.0215339 | 0.9909026 |
| rs7214252 | A | -0.0115982 | 0.00178234 | 7.65E-11 |  | -0.0130255 | 0.0234777 | 0.5790298 |
| rs7250197 | T | 0.0133309 | 0.00205519 | 8.79E-11 |  | -0.0192093 | 0.0269922 | 0.476675 |
| rs725445 | C | -0.0183923 | 0.00148454 | 2.99E-35 |  | 0.112172 | 0.0197777 | 7.07E-09 |
| rs72721921 | A | 0.0120834 | 0.0020148 | 2.01E-09 |  | 0.0163635 | 0.026542 | 0.5375566 |
| rs7306710 | C | -0.0123489 | 0.00147693 | 6.21E-17 |  | 0.0170009 | 0.0194546 | 0.3821869 |
| rs7575451 | G | -0.0102935 | 0.00152413 | 1.44E-11 |  | -0.0182773 | 0.0200543 | 0.3620872 |
| rs7593049 | T | 0.0133747 | 0.00151821 | 1.26E-18 |  | 0.0106708 | 0.0199678 | 0.5930643 |
| rs76895963 | G | 0.0447386 | 0.00625273 | 8.36E-13 |  | -0.0811288 | 0.0836272 | 0.3319845 |
| rs772014 | G | -0.0103903 | 0.00148691 | 2.79E-12 |  | 0.00742961 | 0.0195823 | 0.7043883 |
| rs77485342 | T | 0.0363611 | 0.00542747 | 2.09E-11 |  | -0.13047 | 0.0922954 | 0.07873765 |
| rs7856625 | T | -0.0125486 | 0.00148799 | 3.36E-17 |  | 0.00437457 | 0.0196056 | 0.8234355 |
| rs7928842 | C | -0.0112705 | 0.00147388 | 2.06E-14 |  | 0.0301264 | 0.0194252 | 0.1209283 |
| rs7939128 | G | 0.0186013 | 0.00161007 | 7.12E-31 |  | -0.0240407 | 0.0211962 | 0.2567111 |
| rs7986514 | G | -0.0151305 | 0.00254045 | 2.59E-09 |  | 0.0149273 | 0.033424 | 0.6551616 |
| rs823130 | T | -0.0122699 | 0.0014741 | 8.53E-17 |  | 0.0313648 | 0.0194405 | 0.1066628 |
| rs827987 | T | -0.00927221 | 0.00147482 | 3.24E-10 |  | 0.00772691 | 0.0194264 | 0.6908122 |
| rs9267806 | A | -0.0178657 | 0.00166928 | 9.89E-27 |  | -0.0325517 | 0.0219153 | 0.1374539 |
| rs934075 | A | -0.00979859 | 0.0015608 | 3.43E-10 |  | -0.0235559 | 0.0205392 | 0.2514314 |
| rs9375702 | T | -0.0154941 | 0.00157208 | 6.47E-23 |  | -0.0160369 | 0.020692 | 0.4383212 |
| rs9396861 | A | -0.0102331 | 0.00154898 | 3.94E-11 |  | 0.064574 | 0.0219449 | 0.001627626 |
| rs9469813 | C | -0.0133101 | 0.00223296 | 2.51E-09 |  | 0.0360507 | 0.0292934 | 0.2184439 |
| rs997850 | C | -0.0090347 | 0.00150802 | 2.08E-09 |  | 0.0332911 | 0.0252968 | 0.09408325 |

SNP, single nucleotide polymorphism; MR, Mendelian randomization; HOA, hand osteoarthritis; XO Study, Xiangya Osteoarthritis Study; SE, standard error.

#
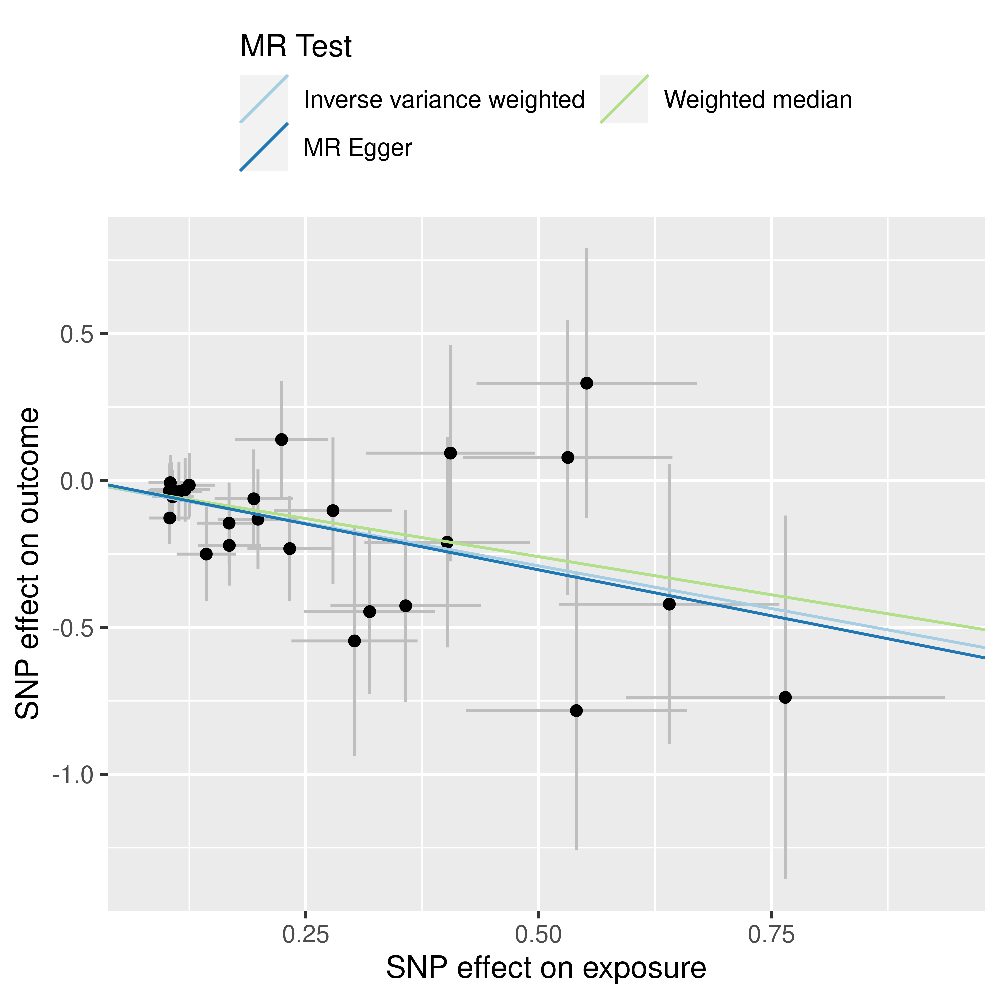
Figure S1. MR scatterplots of the effects of grip strength in incident symptomatic HOA in the XO Study.

SNP, single nucleotide polymorphism; MR, Mendelian randomization; HOA, hand osteoarthritis; XO Study, Xiangya Osteoarthritis Study**.**

#
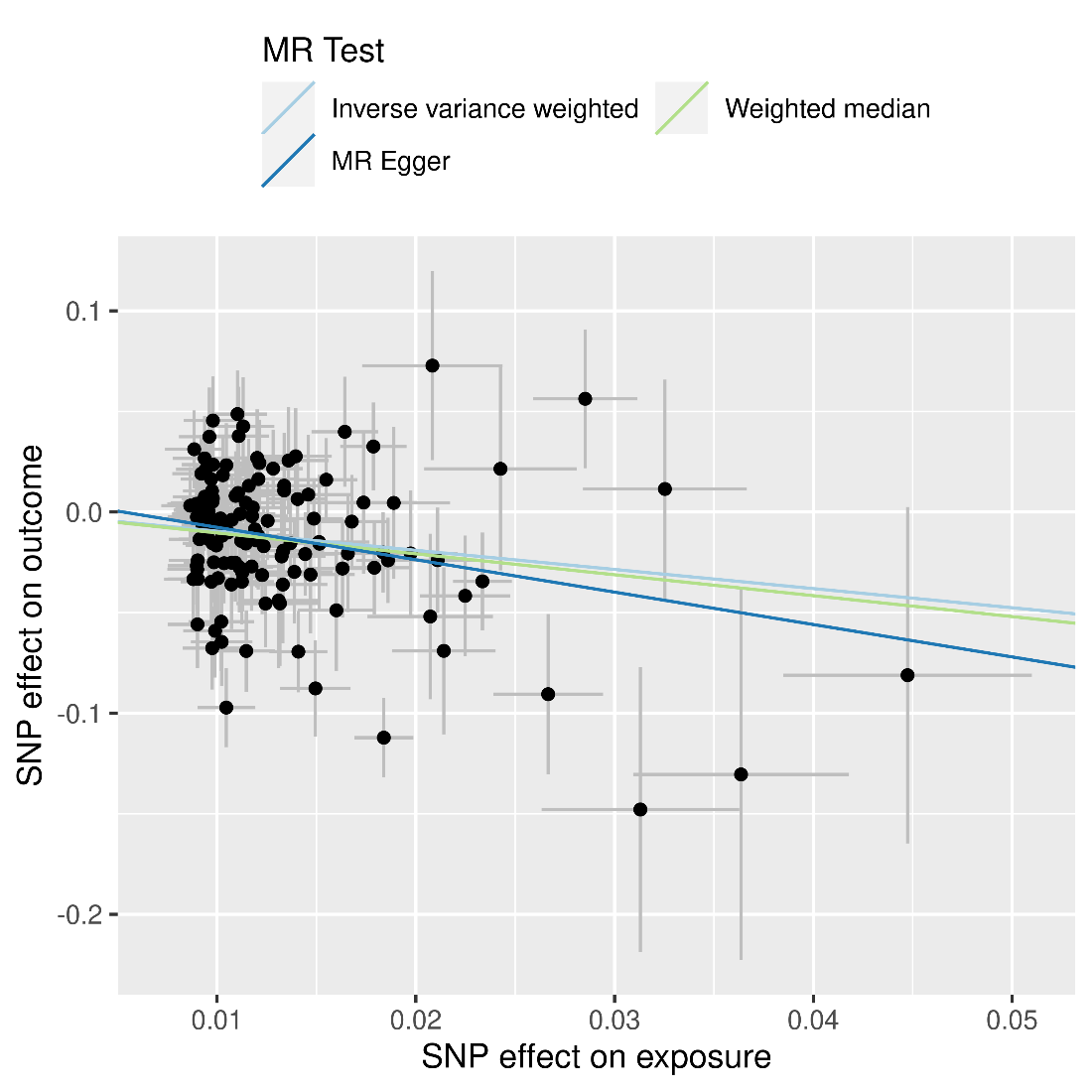
Figure S2. MR scatterplots of the effects of grip strength in incident hospital-diagnosed HOA in the UK Biobank.

SNP, single nucleotide polymorphism; MR, Mendelian randomization; HOA, hand osteoarthritis**.**

# eMethods

**Intra- and inter-reader reliability for HOA**

All hand radiographs were read by a single musculoskeletal researcher (TY; an orthopedic surgeon who was the primary reader). With each new batch of radiographs (n = 50 films), we commingled 5 previously read radiographs to test intra-reader reliability. For assessment of inter-reader reliability, another reader (WZ; a musculoskeletal imaging specialist) scored a selected subset of 30 films independently. Intra-and interrater reliabilities were assessed using kappa statistics with 95% confidence intervals (95% CIs). Radiographic HOA was defined as the presence of a K/L radiographic severity grade of ≥2 in any of the joints of each hand [1]. Using X-ray assessments for diagnosing HOA based on joint evaluation showed excellent intra-reader reliability (kappa) of 0.91 (95% confidence interval [CI], 0.83 to 0.99) and good inter-reader reliability (presence versus absence, kappa of 0.71 [95%CI, 0.45 to 0.96]).

**Intra- and inter-class correlation coefficients for grip strength**

Measurements for grip strength were tested by a single musculoskeletal researcher (TL; an orthopedic surgeon who was the primary investigator). A single investigator measured grip strength on the same subject before and after a one-week interval to assess the intra-rater reliability. For assessment of interrater reliability, two examiners, blind to each other’s test results, independently performed grip strength tests on the same subject. Measurements of grip strength showed excellent reliability of interclass correlation coefficient of 0.97 (95%CI, 0.95 to 0.99) in the left hand and 0.95 (95%CI, 0.91 to 0.97) in the right hand and an intraclass correlation coefficient of 0.90 (95%CI, 0.82 to 0.94) in the left hand and 0.89 (95%CI, 0.80 to 0.94) in the right hand.

**Whole-genome sequencing in the XO Study**

The whole-genome sequencing was done using BGI’s DNBSEQ sequencing technology platform. A total of 2,980 samples that passed quality control (including concentration, sample integrity, and purity) were randomly fragmented by Covaris. The fragmented genomic DNA was then selected by Magnetic beads to an average size of 200-400bp and then end-repaired and 3’ adenylated. Adaptors were ligated to the ends of these 3’ adenylated fragments. The polymerase chain reaction (PCR) process was used to amplify fragments with adaptors, and the Magnetic beads were used to purify PCR products. The double-stranded PCR products were then denatured and circularized by the splint oligo sequence to obtain single-strand circle DNA, which was formatted as the final library. Finally, the library was amplified with phi29 to make DNA nanoball that have more than 300 copies of one molecular, which were then loaded into the patterned nanoarray, and pair-end 100/150 bases reads were generated in the way of combinatorial Probe-Anchor Synthesis.

**Genome-wide association studies (GWASs)**

A total of 2,945 (309 cases) and 2,899 individuals with East Asian ancestry in the XO Study were available to calculate GWAS summary statistics for symptomatic hand osteoarthritis (HOA) and grip strength, respectively. In the UK Biobank, we restricted our GWAS analyses to individuals with European ancestry, resulting in a population of 458,108 individuals available for grip strength. For hospital-diagnosed HOA, we further excluded individuals diagnosed with any musculoskeletal disorders in the control set, which generated a population of 245,671 individuals with 5,626 hospital-diagnosed HOA cases.

To efficiently control for potential case-control imbalance and sample relatedness in the population, we hereby adopted the SAIGE (<https://saigegit.github.io/SAIGE-doc/>) [2] method to calculate summary statistics (i.e., β coefficients and standard errors) for symptomatic HOA. This method contains three steps: 1) Compute sparse genetic relationship matrix (GRM); 2) Fit the null generalized linear mixed model and estimate variance ratio; 3) Perform association tests for each genetic marker. The GRM was estimated using linkage disequilibrium (LD) pruned independent variants (40,840,226 in the XO Study and 376,279 in the UK Biobank). In the XO Study, sex and the first five principal components (PCA) of ancestry were used as fixed effects for grip strength, and sex and the first three PCA were used for symptomatic HOA. In the UK Biobank, age, sex, birth location, batch, and the first 40 PCA were adjusted for both grip strength and hospital-diagnosed HOA. The genomic inflation factors (i.e., lambdas) for symptomatic, hospital-diagnosed HOA, and grip strength from the XO Study and the UK Biobank ranged from 1.019 to 1.159.

**One-sample Mendelian randomization (MR) analyses**

Summary-level data from the above steps were used for one-sample MR analyses [3, 4].

We extracted all SNPs associated with grip strength and then pruned with the pairwise linkage disequilibrium (LD) R^2^>0.001 based on the LD reference panel of East Asian and European ancestry for the XO Study and the UK Biobank, respectively, to assure statistical independence [3, 4].

The inverse-variance weighted (IVW) [4] method was used as the primary approach, which assumes all SNPs are valid instrumental variables (IVs) without horizontal pleiotropy. This method combines the ratio estimates from multiple instruments into a single, pooled estimate, providing the most precise and unbiased estimates. To test the robustness of the primary results and assess potential violations of MR assumptions, we additionally performed weighted median, MR-Egger, and MR pleiotropy residual sum and outlier (MR-PRESSO) analyses, each relying on different assumptions. The weighted median [5] estimator offers protection against invalid instruments, yielding a consistent estimate even up to (but not including) 50% of IVs are invalid. The MR-Egger method [6] provides a consistent estimate under the Instrumental Strength Independent of the Direct Effect (InSIDE) assumption and allows testing of the IV assumptions via the intercept. The MR-PRESSO test [7] identifies and removes horizontal pleiotropic outliers to correct potential directional horizontal pleiotropy and resolve detected heterogeneity. Consistent estimates across these MR approaches strengthen causal inference. In addition, F-statistics were calculated to detect potential weak instrument bias, particularly relevant in a one-sample setting; an F-statistic ≥10 indicates no substantial evidence of weak instrument bias [8].

# REFERENCE

1. Kellgren JH, Lawrence JS. Radiological assessment of osteo-arthrosis. Ann Rheum Dis. 1957;16:494-502. doi:10.1136/ard.16.4.494

2. Zhou W, Nielsen JB, Fritsche LG, Dey R, Gabrielsen ME, Wolford BN, et al. Efficiently controlling for case-control imbalance and sample relatedness in large-scale genetic association studies. Nat Genet. 2018;50:1335-41. doi:10.1038/s41588-018-0184-y

3. Liu X, Tong X, Zou Y, Lin X, Zhao H, Tian L, et al. Mendelian randomization analyses support causal relationships between blood metabolites and the gut microbiome. Nat Genet. 2022;54:52-61. doi:10.1038/s41588-021-00968-y

4. Burgess S, Butterworth A, Thompson SG. Mendelian randomization analysis with multiple genetic variants using summarized data. Genet Epidemiol. 2013;37:658-65. doi:10.1002/gepi.21758

5. Bowden J, Davey Smith G, Haycock PC, Burgess S. Consistent Estimation in Mendelian Randomization with Some Invalid Instruments Using a Weighted Median Estimator. Genet Epidemiol. 2016;40:304-14. doi:10.1002/gepi.21965

6. Bowden J, Davey Smith G, Burgess S. Mendelian randomization with invalid instruments: effect estimation and bias detection through Egger regression. Int J Epidemiol. 2015;44:512-25. doi:10.1093/ije/dyv080

7. Verbanck M, Chen CY, Neale B, Do R. Detection of widespread horizontal pleiotropy in causal relationships inferred from Mendelian randomization between complex traits and diseases. Nat Genet. 2018;50:693-8. doi:10.1038/s41588-018-0099-7

8. Pierce BL, Ahsan H, Vanderweele TJ. Power and instrument strength requirements for Mendelian randomization studies using multiple genetic variants. Int J Epidemiol. 2011;40:740-52. doi:10.1093/ije/dyq151
